# Morning blood pressure surge and target organ damage in newly diagnosed type 2 diabetic patients: a cross sectional study

**DOI:** 10.1186/s12902-015-0068-4

**Published:** 2015-12-03

**Authors:** Johanne M. Lyhne, Esben Laugesen, Pernille Høyem, Simon Cichosz, Jens S. Christiansen, Søren T. Knudsen, Klavs W. Hansen, Troels K. Hansen, Per L. Poulsen

**Affiliations:** Department of Endocrinology and Internal Medicine, Aarhus University Hospital, Nørrebrogade 44, DK-8000 Aarhus C, Denmark; Department of Clinical Medicine, Aarhus University, Aarhus, Denmark; The Danish Diabetes Academy, Odense University Hospital, Odense, Denmark; Medical department, Diagnostic Center, Regional Hospital Silkeborg, Silkeborg, Denmark

**Keywords:** Morning blood pressure surge, Systolic night-day ratio, Ambulatory blood pressure monitoring, Type 2 diabetes, Vascular target organ damage

## Abstract

**Background:**

Type 2 diabetic patients display significantly higher incidence of cardiovascular (CV) events including stroke compared to non-diabetics. Morning blood pressure surge (MBPS) and blunted systolic night-day (SND) ratio have been associated with CV events in hypertensive patients. No studies have evaluated MBPS in newly diagnosed diabetic patients or studied the association with vascular target organ damage at this early time point of the diabetes disease.

**Methods:**

Ambulatory blood pressure monitoring was performed in 100 patients with newly diagnosed type 2 diabetes and 100 age and sex matched controls. MBPS and SND-ratio were calculated. Markers of early vascular target organ damage included pulse wave velocity (PWV), white matter lesions (WML) on brain MRI, and urine albumin/creatinine ratio (UAE).

**Results:**

No significant differences in MBPS were found between diabetic patients and controls. Neither MBPS or SND-ratio were associated with PWV, UAE or WML in the diabetic group independently of age, gender and 24-h systolic blood pressure. 40.2 % of diabetic patients and 25.8 % of controls were classified as non-dippers (*p* = 0.03).

**Conclusion:**

MBPS and SND-ratio are not associated with subclinical markers of vascular target organ damage in our study sample of newly diagnosed type 2 diabetic patients.

## Background

Type 2 diabetic patients exhibit significantly higher cardiovascular (CV) morbidity and mortality compared with non-diabetics [1]. Recent research has suggested the morning blood pressure surge (MBPS) as a new risk marker in ambulatory blood pressure (BP) monitoring [2–5]. It has been suggested that an exaggerated MBPS may be involved in the increased incidence of CV events occurring in the morning, as BP and CV events display parallel diurnal variations [4, 5]. Although MBPS is correlated to the more established systolic night/day (SND) ratio, evaluation of MBPS in clinical studies has yielded conflicting data. Indeed, in previous studies in mainly hypertensive populations a large MBPS has been associated to both increased [4, 6–9] and decreased [3, 10, 11] risk of cerebro- and cardiovascular events.

Only a few studies have examined MBPS as a CV risk factor in type 2 diabetic patients. Both studies included diabetic patients with long duration of diabetes and with hypertension as an isolated risk factor. Eguchi et al. [12] found no association between MBPS and incident cardiovascular events in a 54 months follow-up study, while Hermida et al [13] found that a larger MBPS was associated with lower cardiovascular risk independently of office BP, but not independently of systolic night blood pressure. It remains unknown to what extent MBPS and SND-ratio are independent risk markers in the early phase of the diabetes disease.

The reasons for the contrasting findings are unclear and a better understanding of the pathophysiological mechanisms linking MBPS to CV events is needed. It has been suggested, that MBPS may accelerate vascular injury eventually leading to CV events [14], however only few studies have evaluated this. Carotid femoral pulse wave velocity (PWV), cerebral white matter lesions (WML) and urinary albumin excretion prognosticate CV events and are markers of vascular target organ damage yet their association with MBPS only sparsely investigated.

Therefore, this study was designed to i) assess MBPS and SND-ratio in a sample of recently diagnosed diabetic patients and sex- and age-matched controls and ii) to investigate the association between MBPS and SND-ratio and PWV, urine albumin/creatinine ratio (UAE), and WML in type 2 diabetes.

## Methods

### Subjects and definitions

The study sample has been described in detail previously [15]. 100 patients with type 2 diabetes and 100 control subjects matched individually for sex and age were included in the study. The patients were recruited consecutively from the outpatient specialised clinics at Aarhus University Hospital, Aarhus, Denmark. Inclusion criteria were (i) age > 18 year, (ii) diagnosis of type 2 diabetes according to World Health Organization criteria [16] and (iii) known duration of diabetes less than five years. The control subjects were recruited by advertising in local newspapers, and undiagnosed diabetes was excluded at baseline by fasting glucose and oral glucose tolerance testing. Exclusion criteria for both groups were acute or chronic infectious diseases, end stage renal failure, pregnancy or lactation, prior or current cancer, and contraindications to magnetic resonance imaging (MRI). Clinic blood pressure (BP) was calculated as the average of three measurements. Time and dosage of antihypertensive treatment was unchanged during the study. Body mass index was calculated as weight/height^2^ (kg/m^2^). Smoking was defined as current, previous, or never. The study was approved by the Research Ethics Committee of Central Region, Denmark and by the Danish Data Protection Agency. All patients gave their written informed consent to participate.

### Ambulatory blood pressure monitoring (ABPM)

BP and heart rate were monitored for 24 h using a non-invasive, portable device (Spacelabs 90217, Spacelabs Healthcare, Issaquah, WA) validated according to the British Hypertension Society protocol [17]. Automatic BP recordings were programmed to occur at 20 min interval during day and night. Day/night hours and time of waking up were based on mini-diaries filled out by the participants during ABPM. Recordings with more than three missing hours (maximum of 1 h during night) were excluded from the analyses (3 participants and their corresponding matches, i.e. 6 subjects). Measurements were performed during a day with normal activities at home or at work. Mean awake and sleep levels of SBP and DBP were calculated. Hypertension was diagnosed as 24-h SBP > 130 and/or 24 h diastolic BP (DBP) > 80 mmHg. Dipping-status was based on the reduction in nocturnal blood pressure relative to daytime values. The participants were classified as non-dippers if the sleep SBP decrease was < 10 %.

### Morning Blood Pressure Surge (MBPS)

The most common definitions of MBPS are the sleep-trough surge and the pre-waking surge, in this study MS1 and MS2, respectively. Alternative definitions have been introduced in the literature [2, 18] in this study MS3-MS5. MS1 was defined as the morning BP (the average of the two hours just after waking up) minus the lowest night-time BP (the average of three readings centred on the lowest night-time reading). Two missing values were accepted, but not the first two after waking up. MS2: Morning BP in the two hours just after waking up minus the average of the readings in the two hours just before waking up. Again, two missing values were accepted, but not the last two before waking up. MS3 was calculated as the first BP after waking up minus the last BP before waking up. No missing values were accepted. MS4: Average BP one hour after waking up minus the average of the whole night BP. One missing value was accepted after waking up, but not the first one. No more than one hour missing data accepted during the night. MS5: The average of the two hours after waking up minus the whole-night average.

The clinical perspectives regarding MBPS were discussed by Li et al. [6]. Using two different definitions for MBPS (MS1 and MS2), the authors suggest that a systolic MBPS of <20 mmHg is unlikely to be associated with an increased risk of cardiovascular events in hypertensive patients after accounting for SND-ratio. To evaluate this cut-off, we dichotomised MBPS data (MBPS < 20 mmHg = 0, MBPS ≥ 20 mmHg = 1).

### Markers of vascular target organ damage

Brain MRI was performed with an eight-channel SENSE head coil on a 1,5-T MRI scanner (Achieva, Philips, Best, Netherlands) to obtain axial T2-flair-weighted scans with a slice thickness of 5 mm. WMLs were assessed by a blinded, experienced radiologist on T2-weighted scans and graded 0–2 using Breteler’s scale (0–4 punctate WMLs = 0, >4 punctate WMLs but no confluent lesions = 1, presence of confluent WMLs regardless of number of punctate lesions = 2) PWV was performed using an applanation tonometer (Millar, SPT-301B, Houston, TX) and SphygmoCor equipment and software, version 8.0 (AtCor Medical, Sydney, Australia). The transit time was determined by the intersecting tangent algorithm. The distance between the two arteries was measured directly on the body using a tape measure and the PWV calculated as distance divided by time (m/s). UAE was evaluated by albumin/creatinine ratio in a morning urine sample. MRI data were not available for four participants because of claustrophobia. PWV data were not recordable in four participants because of atrial fibrillation and in three patients due to obesity.

### Statistical analysis

Group differences in continuous variables were assessed using paired *t* tests. Assumption of normal distributions was tested by histograms and Q-Q plots. Skewed data (HbA1c, total cholesterol, triglycerides and urine albumin-to-creatinine ratio) were log-transformed prior to analysis to obtain normal distribution. Categorical data were tested by Chi^2^ test. Baseline data are presented as means ± SD or median (25^th^ percentile; 75^th^ percentile) for skewed data. Associations between morning surge data/systolic night day-ratio and UAE and PWV were assessed by Pearson’s correlation coefficient and by multivariate linear regression analysis. Association with Breteler score was assessed with ordinal logistic regression analysis. In all multivariate analyses, we adjusted for age and sex, and in analyses in the pooled sample additionally for the effect of diabetes (yes/no). Blood pressure levels may confound the association between morning surge/systolic night-day ratio and the three outcomes (PWV, UAE and WML). Accordingly, the effect of including 24-h systolic BP in the analyses with age and sex was also evaluated in supplementary analyses. PWV is also known to be associated with office mean BP and heart rate, and the effect of including these parameters together with age and sex in the analyses with PWV as outcome was also assessed. A two-tailed P value of less than 0.05 was considered statistically significant. All statistical calculations were performed with software from Stata version 11; StataCorp LP, College Station, Texas, USA). Not all morning BP surge parameters could be calculated for all participants due to missing BP data during the night. Accordingly, we did power calculations for different potential sample sizes. With 80 participants in each group, a standard deviation of 10 mmHg and a 5 % α-level, we had 97 % power to detect a difference of 6 mmHg in morning surge between the groups. With 60 participants in each group, a standard deviation of 10 mmHg and a 5 % α-level, we had 90 % power to detect a difference of 6 mmHg.

## Results

Clinical and laboratory characteristics are presented in Table 1 for the 97 participants with data on SND-ratio and their matched control subjects. Diabetic patients were overweight, and the proportion taking antihypertensive and cholesterol-lowering treatment was significantly higher compared with the control group. Consequently, the diabetic group had significantly lower office BP and cholesterol levels than the control group. 24-h, day- and night-time systolic and diastolic blood pressures were not significantly different between the two groups. The diabetic group was characterized by significantly higher urine albumin/creatinine ratio (UAE) and PWV. Breteler score was similar distributed in the two groups.

**Table 1:** Patient characteristics

	Patients with diabetes	Controls	*P*-value
Sex, *n* (Male/female)	97 (50/47)	97 (50/47)	-
Age (years)	58.5 ± 9.9	58.3 ± 9.8	-
Diabetes duration (years) (median, (25^th^;75 ^th^)	1.8; 0.7; 3.2	-	-
Smoking, *n* (Current/previous/never)	21 / 36 / 39	21 / 32 / 44	0.77
Statin use, *n* (Yes/no)	74/22	18/79	<0.001
Antihypertensive treatment, *n* (Yes/no)	60/36	23/74	<0.001
RAAS-inhibitors, *n*	48	11	
Calcium-antagonists, *n*	14	9	
Diuretics, *n*	39	10	
*β*-blockers, *n*	9	7	
BMI (kg/m2)	30.1 ± 4.8	26.1 ± 4.1	<0.001
HbA1c %	6.5 ± 0.6	5.6 ± 0.3	<0.001
HbA1c mmol/mol	48 ± 17	38 ± 20	<0.001
Total cholesterol (mg/dL)	79.2 ± 14.4	100.8 ± 18.0	<0.001
LDL-cholesterol (mg/dL)	41.4 ± 12.6	59.4 ± 18.0	<0.001
HDL-cholesterol (mg/dL)	25.2 ± 5.4	30.6 ± 10.8	<0.001
Triglycerides (mg/dL) (Median, 25th and 75th)	25.2; 19.8; 34.2	21.6; 16.2; 28.8	<0.01
Office measurements (mmHg)			
Systolic BP	125.6 ± 11.9	132.2 ± 15.7	<0.05
Diastolic BP	78.8 ± 7.9	84.0 ± 10.5	<0.01
Heart rate	65.9 ± 9.6	62.2 ± 10.7	<0.05
24-h ABPM (mmHg)			
Systolic BP	125.3 ± 10.8	124.2 ± 12.4	0.49
Diastolic BP	74.0 ± 7.5	75.3 ± 7.4	0.21
Heart rate	73.3 ± 9.5	68.3 ± 8.9	<0.001
Daytime ABPM (mmHg)			
Systolic BP	130.6 ± 11.1	130.5 ± 13.3	0.97
Diastolic BP	78.2 ± 8.0	79.9 ± 8.2	0.12
Heart rate	76.7 ± 10.1	71.8 ± 9.8	<0.01
Nighttime ABPM (mmHg)			
Systolic BP	114.9 ± 11.7	112.0 ± 12.0	0.08
Diastolic BP	65.8 ± 7.7	66.3 ± 7.4	0.62
Heart rate	66.6 ± 9.7	61.5 ± 8.8	<0.001
Pulse wave velocity (m/s)	9.3 ± 2.0	8.0 ± 1.6	<0.001
Urine albumine/creatinine (mg/mmol)			
(Median, (25^th^;75 ^th^))	0.31 (0.21; 0.67)	0.23 (0.15; 0.40)	<0.01
Breteler score, n (0/1/2)	52 / 32 / 10	51 / 32 / 13	0.83

Data are presented as n, mean ± SD, or median (interquartile range)

*Abbreviations*: *BMI* Body Mass Index, *LDL* Low Density Lipoprotein, *HDL* High Density Lipoprotein, *BP* Blood Pressure, *ABPM* Ambulatory Blood Pressure Monitoring

Systolic BP was used for all 5 MBPS calculations in the 200 participants. Missing values were due to removal of the portable recording device < 2 h after waking up (156 calculations) and insufficient night-measurements (27 calculations). Accordingly, 817 out of 1000 calculations were successful. As our inclusion was based on matching data, only matched data was used for comparing diabetic patients and controls and to this end we excluded MS-data without a match. Finally, 79 matched calculations were available for MS3, 68 for MS4 and 63 for MS1, MS2 and MS5.

**Fig. 1:**
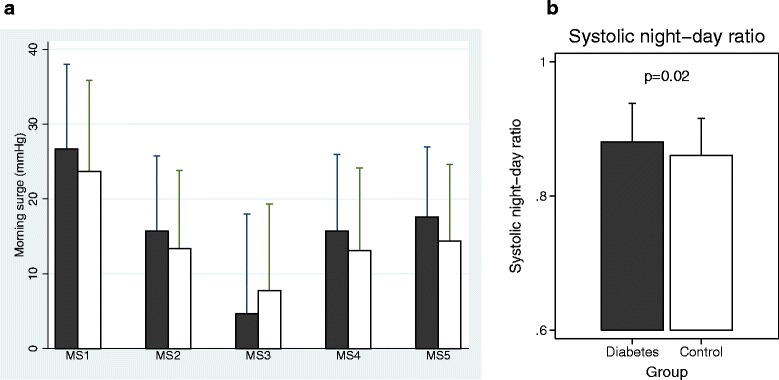
Panel **a**: Morning blood pressure surge, MS1-MS5 in patients with diabetes (black columns) and controls (white columns) matched for age and gender. Mean ± SD. MS1: Average of 2 h after waking minus average of lowest night-hour. MS2: Average of 2 h after waking minus average of 2 h before waking. MS3: First BP after waking minus last BP before waking. MS4: Average of 1 h after waking minus average night BP. MS5: Average 2 h after waking minus average night. (MS1: 27.5 vs. 24.6 mmHg; *p* = 0.13), (MS2: 16.3 vs. 14.0 mmHg; *p* = 0.20), (MS3: 4.7 vs. 7.8 mmHg; *p* = 0.11), (MS4: 16.0 vs. 13.6 mmHg; *p* = 0.21), (MS5: 18.2 vs. 15.3 mmHg; *p* = 0.07). Panel **b**: Systolic night/day-ratio in patients with diabetes (black column) and controls (white column)

No significant differences were found for any of the five definitions of MBPS when comparing the diabetic group and the control group, Fig. 1a (MS1: 27.5 ± 11.2 vs. 24.6 ± 12.2 mmHg; *p* = 0.13), (MS2: 16.3 ± 10.3 vs. 14.0 ± 11.4 mmHg; *p* = 0.20), (MS3: 4.7 13.3 ± vs. 7.8 ± 11.6 mmHg; *p* = 0.11), (MS4: 16.0 ± 10.4 vs. 13.6 ± 11.2 mmHg; *p* = 0.21), (MS5: 18.2 ± 9.3 vs. 15.3 ± 10.1 mmHg; *p* = 0.07). The SND-ratio was significantly higher in the diabetic group than in the control group, Fig. 1b (0.88 vs. 0.86; *p* = 0.02). 39 patients with diabetes (40.2 %) and 25 of the controls (25.8 %) were classified as non-dippers, *p* = 0.03.

Univariate and multivariate regression analyses between MBPS and the subclinical markers PWV, UAE and WML are presented in Table 2 for MS1 and MS2. No independent associations between MBPS indices and WML, PWV, or lnUAE were found after adjustment for age and sex. Further adjustment for 24-h SBP and diabetes in the analyses with lnUAE, WML and PWV and for mean arterial pressure and heart rate in the analyses with PWV in fully adjusted models did not change the results in the total study group or in the diabetic or control group. Analysis regarding MS3-MS5 showed similar results.

**Table 2:** Beta estimates for univariate and multivariate regression analyses of the association between MBPS and subclinical cardiovascular risk markers

Morning surge	Pulse wave velocity	Urinary albumin excretion	White matter lesions
	m/s / mmHg	Ln (mg/mmol) / mmHg	OR/mmHg
	Mean ± SE	Mean ± SE	Mean ± SE
All			
MS1			
Unadjusted	0.016 ± 0.010; *p* = 0.10	0.018 ± 0.007; *p* = 0.011	0.007 ± 0.014; *p* = 0.62
Adjusted	−0.002 ± 0.010; *p* = 0.81	0.014 ± 0.007; *p* = 0.053	−0.002 ± 0.015; *p* = 0.90
MS2			
Unadjusted	0.006 ± 0.016; *p* = 0.70	0.006 ± 0.009; *p* = 0.51	0.005 ± 0.013; *p* = 0.69
Adjusted	−0.006 ± 0.011: *p* = 0.63	0.005 ± 0.008; *p* = 0.56	−0.001 ± 0.016; *p* = 0.93
Patients			
MS1			
Unadjusted	−0.017 ± 0.025; *p* = 0.51	0.019 ± 0.012; *p* = 0.11	−0.013 ± 0.022; *p* = 0.56
Adjusted	−0.027 ± 0.020; *p* = 0.18	0.021 ± 0.012; *p* = 0.08	−0.033 ± 0.024; *p* = 0.18
MS2			
Unadjusted	−0.011 ± 0.027; *p* = 0.68	−0.002 ± 0.013; *p* = 0.86	0.003 ± 0.023; *p* = 0.91
Adjusted	−0.020 ± 0.022; *p* = 0.38	0.002 ± 0.014; *p* = 0.89	−0.013 ± 0.027; *p* = 0.64
Controls			
MS1			
Unadjusted	0.028 ± 0.016; *p* = 0.08	0.012 ± 0.007; *p* = 0.08	0.022 ± 0.019; *p* = 0.26
Adjusted	0.018 ± 0.014; *p* = 0.20	0.009 ± 0.007; *p* = 0.17	0.018 ± 0.020; *p* = 0.36
MS2			
Unadjusted	0.007 ± 0.019; *p* = 0.71	0.007 ± 0.008; *p* = 0.41	0.008 ± 0.021; *p* = 0.72
Adjusted	0.006 ± 0.016; *p* = 0.72	0.008 ± 0.008; *p* = 0.32	0.004 ± 0.022; *p* = 0.86

*All* Multivariate analyses adjusted for age, gender and diabetes, *Diabetes/controls* Adjusted for age and gender, *MS* morning surge

Participants with a surge above 20 mmHg in the MBPS parameters used by Li et al. (MS1 and MS2) did not show increased PWV, UAE or WML when compared to participants with a surge below 20 mmHg. However, MS5 above 20 mmHg was significantly associated to UAE (*β* = 0.42 ± 0.17; *p* = 0.02) even when adjusting for age, sex, diabetes mellitus and 24-h systolic BP (*β* = 0.37 ± 0.17; *p* = 0.03). This might be a chance finding rather than an actual validation of the threshold, as none of the other MS-calculations showed the same tendency.

As previous studies on MBPS primarily have focused on hypertensive patients, we divided the two groups into hypertension/normotension based on ABPM data (normotension: <130/80 mmHg on 24-h ABPM) and repeated all univariate and multivariate analyses. In pooled data on all normotensive participants (n = 91) MS1 was significantly associated with lnUAE in univariate and multivariate analyses including age, gender, 24-h SBP and diabetes (*β* = 0.02, *p* = 0.01 and *β* = 0.02, *p* = 0.04, respectively). No association was seen when normotensive diabetic patients were evaluated separately. MS1 was significantly associated with lnUAE in normotensive control subjects (n = 47) in both univariate and multivariate analyses including age, gender and 24-h SBP (*β* = 0.02, *p* = 0.01 and *β* = 0.02, *p* = 0.02, respectively) as were MS4 and MS5 (MS4; *β* = 0.03, *p* = 0.01, MS5; *β* = 0.03, *p* = 0.02 and MS4; *β* = 0.03, *p* = 0.01, MS5; *β* = 0.02, *p* = 0.03, respectively). No association was found to WML or PWV for normotensive participants. MS4 was significantly associated with PWV in a small (n = 24) subgroup of isolated hypertensive controls even with adjustments for age, sex, 24-h SBP and mean arterial pressure and heart rate at time of measuring (*p* = 0.01) (data not shown).

Finally, as antihypertensive treatment (AHT) could be hypothesized to affect the MBPS we divided the groups according to AHT (yes/no). No statistically significant associations between MBPS measurements and PWV, WML or lnUAE in either treated or untreated patients were found after multivariate adjustments including age, gender, diabetes, and 24-h SBP (data not shown).

The same statistical analyses were completed for associations between SND-ratio and PWV, lnUAE, and WML, see Table 3. No independent associations between SND-ratio and WML, PWV, or lnUAE were found after adjustment for age and sex. In the normotensive group (n = 109), SND-ratio was significantly associated with PWV in unadjusted and adjusted models (*β* = 8.04 ± 2.79, *p* = 0.01 and *β* = 6.31 ± 2.48, *p* = 0.01, respectively). The association remained in a fully adjusted model with further adjustment for mean arterial pressure and heart rate (*β* = 5.40 ± 2.27, *p* = 0.02). When evaluated separately, the association was significant in normotensive diabetics (n = 55) in univariate analysis (*β* = 10.27 ± 4.53, *p* = 0.03), but lost in a fully adjusted model. In the normotensive controls (n = 54), an association was seen only in the fully adjusted model (*β* = 5.43 ± 2.69, *p* = 0.049). All groups in AHT were associated with PWV in univariate analyses, but associations were lost when adjusting for age and gender (data not shown). No relation was found to either lnUAE or WML (Table 3).

**Table 3:** Unadjusted/adjusted p-values for univariate end multivariate regression analyses of the association between systolic night/day-ratio and subclinical risk markers

Systolic night-day ratio	Pulse wave velocity (m/s)	Urinary albumin excretion (Ln (mg/mmol))	White matter lesions (Breteler score)
All (176)	0.049 / 0.23	0.72 / 0.77	0.36 / 0.53
Patients with diabetes	0.08 / 0.13	0.86 / 0.99	0.95 / 0.99
Controls	0.45 / 0.85	0.59 / 0.75	0.29 / 0.46
All, normotensive	0.005 / 0.012	0.48 / 0.96	0.31 / 0.27
DM, normotensive	0.027 / 0.09	0.45 / 0.60	0.75 / 0.85
Controls, normotensive	0.38 / 0.10	0.36 / 0.38	0.13 / 0.24
All, hypertensive	0.45 / 0.40	0.84 / 0.72	0.90 / 0.83
DM, hypertensive	0.89 / 0.68	0.33 / 0.31	0.55 / 0.97
Controls, hypertensive	0.06 / 0.13	0.77 / 0.67	0.62 / 0.56

Adjusted for age and gender (and diabetes in pooled data)

*DM* Patients with diabetes

## Discussion

In this study we assessed MBPS in recently diagnosed type 2 diabetic patients compared with a gender- and age-matched control group, and studied the association between MBPS, SND-ratio and markers of vascular target organ damage. The MBPS indices were of similar magnitude in the two groups regardless of methods of calculation. This is opposed to data presented by Afsar et al. [19] who found higher MBPS in diabetic patients and also in contrast with Ayala et al. [20] who reported diabetic patients had significantly lower MBPS than non-diabetics. The background for the diverging results are unclear, but may relate to difference in diabetes duration, age, HbA1c-level, and number and type of antihypertensive drugs in the different studies. Furthermore, we found that the SND-ratio and the prevalence of non-dippers were significantly higher in the diabetic patients. This is in line with previous studies in diabetic patients [21–23]. Afsar et al. [23] studied 96 newly diagnosed type 2 diabetic patients and found a prevalence of 56.3 % non-dippers, which is higher than in our study. Afsar et al. used fixed time intervals for sleep periods and did not include a control group. All the included patients had essential hypertension, which also might influence the result.

Previous studies have reported data suggesting an association between MBPS and perturbed vascular function. Marfella et al. reported increased MBPS was associated with markers on carotid plaque instability in hypertensive patients [24] and Yoda et al. found that increased MBPS was associated with endothelial dysfunction in Japanese type 2 diabetic patients with long disease duration [14]. In our study, none of the five MBPS measurements were independently associated with PWV, WML or UAE. PWV is a marker of structural changes in the large vessel indication deteriorating vessel wall elasticity [25] whereas WML and UAE are primarily manifestations of changes in small vessel in the brain and kidneys [26, 27]. The three markers are previously shown to independently predict future cerebrovascular events in hypertensive and general populations [26] and cardiovascular events in diabetic populations with long duration of diabetes [28–30]. A blunted SND-ratio has also been identified as an independent risk marker of target organ damage and cardiovascular disease in patients with long duration of diabetes [31, 32]. The lack of association between SND-ratio and WML, PWV and lnUAE in diabetic patients in this study suggests that SND-ratio might reflect other aspects of cardiovascular risk than these parameters or that a possible association have not emerged this early in the time course of the diabetic disease. Yet, this remains speculative as our data are cross-sectional and accordingly causality cannot be inferred. However, our results are in line with the longitudinal study by Eguchi et al. [12] who followed 300 Japanese patients with long duration of diabetes for 54 months and found no independent ability of MS1 or dipping patters to predict future cardiovascular events. In the 5.4 year follow-up study of 607 patients with type 2 diabetes conducted by Hermida et al. [13], increased MBPS was significantly associated with lower risk of cardiovascular disease, but the association was lost when including systolic night BP in cox-regression models. The complex interplay between MBPS and SND-ratio seems to be more closely related in diabetic patients than in hypertensive patients, as no studies yet have shown an independent association between MBPS and cardiovascular risk factors in diabetic patients after adjusting for mean night systolic BP. This study suggests, that newly diagnosed type 2 diabetic patients could be added to this conclusion; however further clarification must await longitudinal data.

Previous studies in non-diabetic hypertensive patients have found associations between MBPS and cerebro- and cardiovascular morbidity, especially incidence of stroke [4–9, 33]. When isolating the hypertensive subgroups of the control population we found no association between MBPS or SND-ratio and subclinical risk factors of target organ damage. The only exception was a significant association between MS4 and PWV (*p* < 0,01 fully adjusted). As the group consisted of only 24 hypertensive controls, the multivariate analysis is prone to over-fitting and can at best be interpreted as hypothesis generating. MS1 was associated with lnUAE in pooled data on normotensive participants as were MS1, MS4 and MS5 in the subgroup of normotensive control subjects. This might indicate a linkage between the morning urine and the MBPS, but needs further investigation.

The significant association between SND-ratio and PWV in normotensive participants extends previous reports of hypertensive patients [34] and might indicate early onset of arterial stiffening in non-dippers even in the normotensive BP range. However, when evaluated separately, significance was limited to normotensive controls and only in an adjusted model. Hence, the association seems to be sensitive to influence from covariates, and these aspects should be further evaluated. Jennersjö et al. [35] studied 663 patients with long duration of type 2 diabetes (34.7 % non-dippers) and found non-dipping independently associated with increased PWV. Our results in normotensive diabetic patients tend to go in the same direction, but are insignificant in multivariate analyses. Furthermore, our results indicate, that in a normotensive population regardless of diabetes, SND-ratio might contain more valuable information than MBPS.

Our study has limitation that should be observed when interpreting the results. Due to the cross sectional study design we cannot infer causality regarding the association between MBPS and the three outcomes; PWV, UAE and WML. Furthermore, the diabetic patients had a relatively low PWV and UAE, and the range was quite narrow for both. Thus the chance of finding a signal is reduced and our results may not be applicable in other populations. Lastly, the lack of the statistical difference in the subgroups might be a consequence of low statistical power.

## Conclusion

None of the five definitions of morning blood pressure surge or the systolic night-day ratio were associated with early markers of vascular target organ damage in our study sample of newly diagnosed type 2 diabetes patients. Even though previous studies have found valuable information regarding cardiovascular risk in MBPS in hypertensive patients and SND-ratio in diabetes patients, this study indicates, that these ambulatory blood pressure measurements are not independently associated with PWV, UAE, or WML at early time point of the diabetic disease.
